# Long non-coding RNA MIAT promotes gastric cancer proliferation and metastasis via modulating the miR-331-3p/RAB5B pathway

**DOI:** 10.3892/ol.2020.12219

**Published:** 2020-10-13

**Authors:** Xiao-Mei Li, Yan-Yan Jiao, Bao-Hong Luan, Hong-Xia Wu, Rong-Rong Wang, Jie Zhong

**Affiliations:** 1 Department of Oncology, Qing Dao Cheng Yang People's Hospital, Qingdao, Shandong 266109, P.R. China; 2 Department of Interventional Radiography, Qing Dao Cheng Yang People's Hospital, Qingdao, Shandong 266109, P.R. China

**Keywords:** gastric cancer, long non-coding RNA MIAT, microRNA-331-3p, Ras-related protein Rab-5B

## Abstract

Gastric cancer (GC) remains a threat to the health of the global population. The present study investigated the effects and mechanisms of the long non-coding RNA myocardial infarction associated transcript (MIAT) on the proliferation, apoptosis and metastasis of GC (HGC-27 and AGS) cells. The expression levels of MIAT, micoRNA (miR)-331-3p and RAB5B mRNA were analyzed using reverse transcription-quantitative PCR analysis. Cell growth, apoptosis, migration and invasion were measured using 5-ethynyl-2′-deoxyuridine, flow cytometry, wound healing and Transwell assays, respectively. A luciferase assay was used to determine whether miR-331-3p targeted MIAT and RAB5B. The results indicated that MIAT levels *were significantly upregulated in GC tissues and cells, correlated with* RAB5B levels *and inversely associated with* miR-331-3p levels. MIAT *overexpression promoted proliferation and metastasis, and inhibited the apoptosis of GC cells*. MIAT knockdown *had the opposite effect on GC cells*. The rescue experiments revealed that the effects of MIAT knockdown on the biological behaviour of GC cells were attenuated by RAB5B overexpression. These data suggest that MIAT *promotes* GC progression via modulating miR-331-3p/RAB5B pathway.

## Introduction

Gastric cancer (GC) is a common cancer worldwide and ranks third among the leading causes of cancer-associated mortalities. Incidence rates are markedly elevated in Eastern Asia (incidence rates are 24.7 per 100,000), when compared with Northern America (incidence rates are 8.4 per 100,000) and Northern Europe (incidence rates are 9.3 per 100,000) (1). Targeted therapy with biomarkers for advanced GC has developed rapidly in recent years (2). Due to late diagnosis, patients with extensive invasion and metastasis have poor prognoses (3). Even after a complete resection, recurrence occurs in ~50% of patients (4). As the molecular mechanisms underlying the metastasis and recurrence of GC have not been fully clarified, identifying key GC-promoting molecules may contribute to the understanding of GC pathogenesis and identification of new therapeutic targets.

Long non-coding RNAs (lncRNAs) are a type of RNA transcript that have >200 nucleotides and are not translated into proteins (5). The myocardial infarction associated transcript (MIAT) is first identified to play a role in the pathogenesis of myocardial infarction (6). Recent studies have reported that MIAT is upregulated in several types of cancers including papillary thyroid (7), lung (8) and colorectal cancer (9). However, the underlying molecular mechanism of MIAT in GC remains largely unknown.

MicroRNAs (miRNAs) are 20–23 nucleotides in length and serve a negative regulatory role by binding to the 3′untranslated region (UTR) of target mRNAs, which results in inhibition of mRNA translation or promotion of mRNA degradation (10). lncRNAs can serve as sponges of miRNAs and reduce their regulatory effects on target mRNAs (11). miR-331-3p serves as a potential tumour suppressor in multiple types of human cancers, including pancreatic (12), ovarian (13) and colorectal cancer (14). miR-331-3p has also been demonstrated to inhibit GC cell growth (15). Here, it was speculated that MIAT functions via targeting miR-331-3p.

Ras-related protein Rab-5B (RAB5B), an isoform of RAB5 (16). High expression of RAB5B is associated with cancer progression and poor prognoses in numerous cancer types, including pancreatic (17), breast (18) and ovarian cancer (19). However, the function of RAB5B in GC is yet to be elucidated.

The present study investigated the effects and mechanisms of the lncRNA MIAT on the proliferation, apoptosis and metastasis of GC cells.

## Materials and methods

### 

#### The Cancer Genome Atlas (TCGA) database

Tissues samples in TCGA database (https://cancergenome.nih.gov/) were divided into two groups (MIAT high expression group: n=25; MIAT low expression group: n=194; cut-off=9.96). A survival curve was generated to analyze the association between MIAT expression and the overall survival of patients with GC patients. A log-rank test was used to compare survival times between two groups. The result was considered statistically significant if P<0.05.

#### Patient samples and cell lines

GC tissue samples and paired normal tissue samples were obtained from 47 patients who underwent surgery between March 2017 and March 2018 at Chengyang People's Hospital (Qingdao, Shandong, China) with written informed consent. All samples were frozen in liquid nitrogen and stored at −80°C until use. The present study was approved by the Ethics Committee of Chengyang People's Hospital. GC tissues were fixed in 4% buffered paraformaldehyde for 24 h at 4°C, embedded in paraffin and then sectioned to 5 µm. These sections were stained with hematoxylin for 15 min and eosin for 5 min at 25°C. Samples were examined under a light microscope at a magnification of ×400. The human gastric epithelial mucosa cell line GES-1 and GC cell lines HGC-27, AGS, MKN45 and NCI-N87 were purchased from Nanjing Keygen Biotech Co., Ltd. HGC-27, AGS, MKN45 and NCI-N87 cells were maintained in RPMI-1640 with 10% foetal bovine serum (FBS), 100 U/ml penicillin and 100 µg/ml streptomycin (HyClone; GE Healthcare Life Sciences). GES-1 cells were cultured in Dulbecco's modified Eagle's medium (DMEM) with FBS, 100 U/ml penicillin and 100 µg/ml streptomycin (HyClone; GE Healthcare Life Sciences). All cells were cultured in a humidified chamber with 5% CO_2_ at 37°C.

#### Transfection

pcDNA 3.1-MIAT, pcDNA 3.1-RAB5B, pcDNA 3.1-negative control (pcDNA 3.1-NC), small interfering RNA (si)-MIAT-1, si-RAB5B and negative control siRNA (si-NC) were generated by Shanghai Gene Pharma Company. miR-331-3p mimics, inhibitors and their respective negative controls were purchased from Guangzhou RiboBio Co., Ltd. The siRNA, miR-331-3p mimics and inhibitor sequences are listed in Table I. HGC-27, AGS and GES-1 cells were transfected using Lipofectamine 2000 transfection reagent (Invitrogen; Thermo Fisher Scientific, Inc.) at 37°C, and the fresh medium was changed after 6 h. The final concentration for transfection was 50 nM. HGC-27, AGS and GES-1 cells were harvested at 48 h after transfection.

#### Reverse transcription-quantitative PCR (RT-qPCR) analysis

Total RNA from all cultured cells lines and human tissue was extracted using TRIzol according to the manufacturer's instructions (Invitrogen; Thermo Fisher Scientific, Inc.). cDNA was synthesized from 1 µg total RNA using the PrimeScript^®^ RT reagent kit (Takara Bio, Inc.) according to the manufacturer's instructions. qPCR was performed in an ABI 7500 instrument (Applied Biosystems; Thermo Fisher Scientific, Inc.) using SYBR-Green Real-Time PCR Master Mix (Takara Bio, Inc.). Amplification conditions were 94°C for 7 min, followed by 40 cycles of 95°C for 15 sec, and 60°C for 30 sec. Relative expression levels were quantitated using the 2^−ΔΔCq^ method (20) with β-actin and U6 as reference control. Primer pairs are listed in Table II.

#### Cell Counting Kit (CCK)-8 assay

The AGS and HGC-27 cells without transfection (5×10^3^ cells/well) were cultivated in RPMI-1640 complete medium with 10% FBS or serum-free medium at 37°C for 1, 2 and 3 days. Then, according to the manufacturer's instructions, CCK-8 solution (10 µl; Dojindo Molecular Technologies, Inc.) was supplemented into each well for incubating another 2 h. A microplate reader (BioTek Instruments, Inc.) was used for examining the absorbance at 450 nm. The GES-1 cells (5×10^3^ cells/well) were cultivated in DMEM medium with 10% FBS at 37°C for 1, 2 and 3 days. The next steps were similar to the AGS and HGC-27 cells.

#### Cell proliferation assay

The HGC-27 and AGS cells at 48 h post transfection were seeded into 24-well plates (2×10^4^) and cultured with 5-ethynyl-2′-deoxyuridine (EdU; 50 µM; Guangzhou RiboBio Co., Ltd.) at 37°C for 2 h. The cells were fixed in 4% paraformaldehyde at 25°C for 30 min and incubated with glycine at 37°C for 4 h. After being submerged into the Apollo reaction cocktail (deionized water 469 µl, Apollo reaction buffer 25 µl, Apollo catalytic solution 5 µl, Apollo 567 fluorescent dye solution 1.5 µl and Apollo buffer additive 5 mg) at 37°C for 30 min in the dark, the cells were washed with 0.5% Triton X-100. Cell nuclei were stained using Hoechst 33342 at 37°C for 30 min. Cells were then imaged using a Nikon fluorescence microscope (Eclipse Ti2-U; Nikon Corporation; magnification, ×400).

#### Cell apoptosis analysis

Apoptosis analysis was conducted by flow cytometry using the Annexin V/propidium iodide kit (Nanjing KeyGen Biotech Co., Ltd). The HGC-27, AGS and GES-1 cells at 48 h post transfection were detached from the plate using EDTA-free trypsin, gathered into a cell suspension with 400 µl Annexin binding buffer at a concentration of 10^6^ cells/ml. Then, 5 µl Annexin V-FITC and 10 µl propidium iodide were added, mixed and incubated for 15 min in the dark at room temperature. Cell apoptosis was tested using flow cytometry (A60-Micro, Apogee, UK and BD Accuri C6 Plus; BD Biosciences). The FlowJo software (version 10.0.6; FlowJo, LLC) was applied for data analysis.

#### Wound healing assay

The HGC-27, AGS and GES-1 cells at 48 h post transfection were seeded in a 6-well plate (3×10^5^). Subsequently, monolayer cells were scratched with a 200 µl pipette tip to generate an artificial wound. After the cells were cultured at 37°C for 24 h in serum-free medium, the wound healing rate was calculated. Cells were imaged under a light microscope at a magnification of ×100. Scratch healing was observed at the same location at the 0 and 24 h. HGC-27, AGS and GES-1 cells migration was assessed by calculating scratch width using the formula: The relative scratch width=(number of cells at T24-number of cells at T0)/number of cells at T0 ×100%, where T0 was 0 h and T24 was 24 h.

#### Invasion assay

The HGC-27, AGS and GES-1 cells at 48 h post transfection in serum-free medium (3×10^5^) were added to the upper Transwell chambers which were pre-coated with Matrigel at 37°C for 60 min (BD Biosciences). The bottom chamber contained medium with 10% FBS. After incubation at 37°C for 24 h, the cells were stained with 0.1% crystal violet at 37°C for 30 min. Cell invasion was quantified by counting the number of cells in 5 random fields under a light microscope (magnification, ×100).

#### Luciferase reporter assay

Bioinformatics databases Starbase v3.0 (http://starbase.sysu.edu.cn/index.php) and TargetScanHuman Release 7.2 (http://www.targetscan.org/vert_72/) were used to identify the potential target sequences of miR-331-3p. HGC-27 and AGS cells were seeded into 24-well plates at a density of 6×10^4^ cells per well, followed by cotransfection with MIAT 3′UTR wild-type (wt) or MIAT 3′UTR mutant type (mut) [RAB5B 3′UTR (wt) or RAB5B 3′UTR (mut)] and the miR-331-3p mimics or mimics NC using Lipofectamine 2000 (Invitrogen; Thermo Fisher Scientific, Inc.). The wt and mut containing the putative binding site of miR-331-3p were cloned and established in the firefly luciferase-expressing pMIRREPORT vector (Obio Technology; http://www.obiosh.com/kyfw/xb/sygsmjc/676.html?1574909908). Luciferase activity was detected 48 h after transfection using the dual-luciferase reporter gene assay kit (Promega Corporation) and *Renilla* luciferase activity was used as the normalization.

#### Western blotting

The HGC-27, AGS and GES-1 cells at 48 h post transfection were lysed using ice-cold radioimmunoprecipitation assay (RIPA) lysis buffer (Beyotime Institute of Biotechnology). Protein concentration was quantified using an Enhanced BCA Protein Assay kit (Beyotime Institute of Biotechnology). The total protein extract (40 µg/lane) was separated via 10% SDS-PAGE and transferred to nitrocellulose membranes. The membranes were blocked with 5% non-fat milk for 1 h at room temperature. After blocking, the membranes were incubated with the relevant antibodies for overnight at 4°C. The following antibodies were used in the present study: Bax (cat. no. 2774; 1:1,000; Cell Signaling Technology), Bcl-2 (cat. no. 4223; 1:1,000, Cell Signaling Technology), cleaved caspase-3 (cat. no. 9661; 1:1,000; Cell Signaling Technology), RAB5B (cat. no. ab72907; 1:1,000; Abcam) and β-actin (cat. no. 4967; 1:2,000; Cell Signaling Technology). The following day, the membranes were incubated with horseradish peroxidase-conjugated secondary antibody (cat. no. 7074; 1:5,000; Cell Signaling Technology) for 2 h at room temperature. Proteins were normalized to β-actin levels. Finally, bands were detected by enhanced chemiluminescence (General Electric Company, Inc.) and quantified using ImageJ software (National Institutes of Health).

#### Statistical analysis

Data analysis was performed using GraphPad Prism 7 (GraphPad Software, Inc.). All results were reported as the mean ± standard deviation (SD) of three independent experiments. Differences were assessed using paired Student's t-test or one-way ANOVA followed by Tukey's multiple comparisons test. Pearson's correlation was used to calculate the correlation between expression of MIAT and miR-331-3p (miR-331-3p and RAB5B; MIAT and RAB5B). Survival analysis was performed using the R package ‘survival’ (version 3.6.1) (21,22) and the Kaplan-Meier curve method. A log-rank test was used to compare survival times between two groups. The result was considered statistically significant if P<0.05.

## Results

### 

#### MIAT expression is increased in GC tissues and cell lines

**Figure 1.**
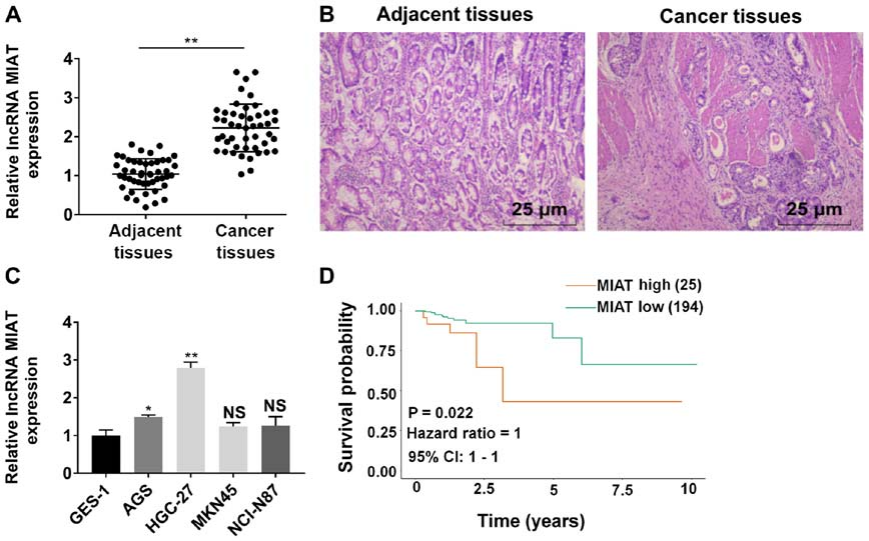
MIAT is upregulated in gastric cancer (GC) tissues and cell lines. (A) RT-qPCR analysis of MIAT expression in GC tissues and corresponding adjacent tissues (n=47). **P<0.01 vs. adjacent tissues group. n=3. (B) The tissue type of gastric cancer was identified as adenocarcinoma. (C) RT-qPCR analysis of MIAT expression in GC cell lines (HGC-27, AGS, MKN45 and NCI-N87) and the gastric epithelial mucosa cell line GES-1. (D) Kaplan-Meier analysis was used to analyze association between MIAT expression and overall survival of patients with GC. n=3. *P<0.05, **P<0.01 vs. the GES-1 group. GC, gastric cancer; RT-qPCR, reverse transcription-quantitative PCR; MIAT, myocardial infarction associated transcript; NS, not significant.

RT-qPCR results revealed that MIAT expression was significantly increased in GC tissue samples (adenocarcinoma) as compared with paratumor tissues (Fig. 1A). Representative staining images are displayed in Fig. 1B. The high expression of MIAT was correlated with late TNM stage and lymph node involvement (Table III). In Fig. 1C, MIAT expression was detected in GES-1 cells and four GC (AGS, HGC-27, MNK45 and NCI-N87) cell lines. The expression of MIAT in AGS, HGC-27, MNK45 and NCI-N87 cells was higher compared with GES-1 cells. Among the four cell lines, MIAT expression was significantly higher in AGS and HGC-27 cells, compared with GES-1 cells. Therefore, AGS and HGC-27 cells were selected to perform subsequent experiments. The expression of MIAT in HGC-27 cells was higher compared with in AGS cells (Fig. S1A). As expected, the proliferation, migration and invasion ability of HGC-27 cells was higher compared with AGS cells (Fig. S1B and C). Kaplan-Meier analysis revealed that high expression of MIAT was associated with poor overall survival time based on data from TCGA database (Fig. 1D).

#### MIAT promotes HGC-27 and AGS cell proliferation and inhibits apoptosis

**Figure 2.**
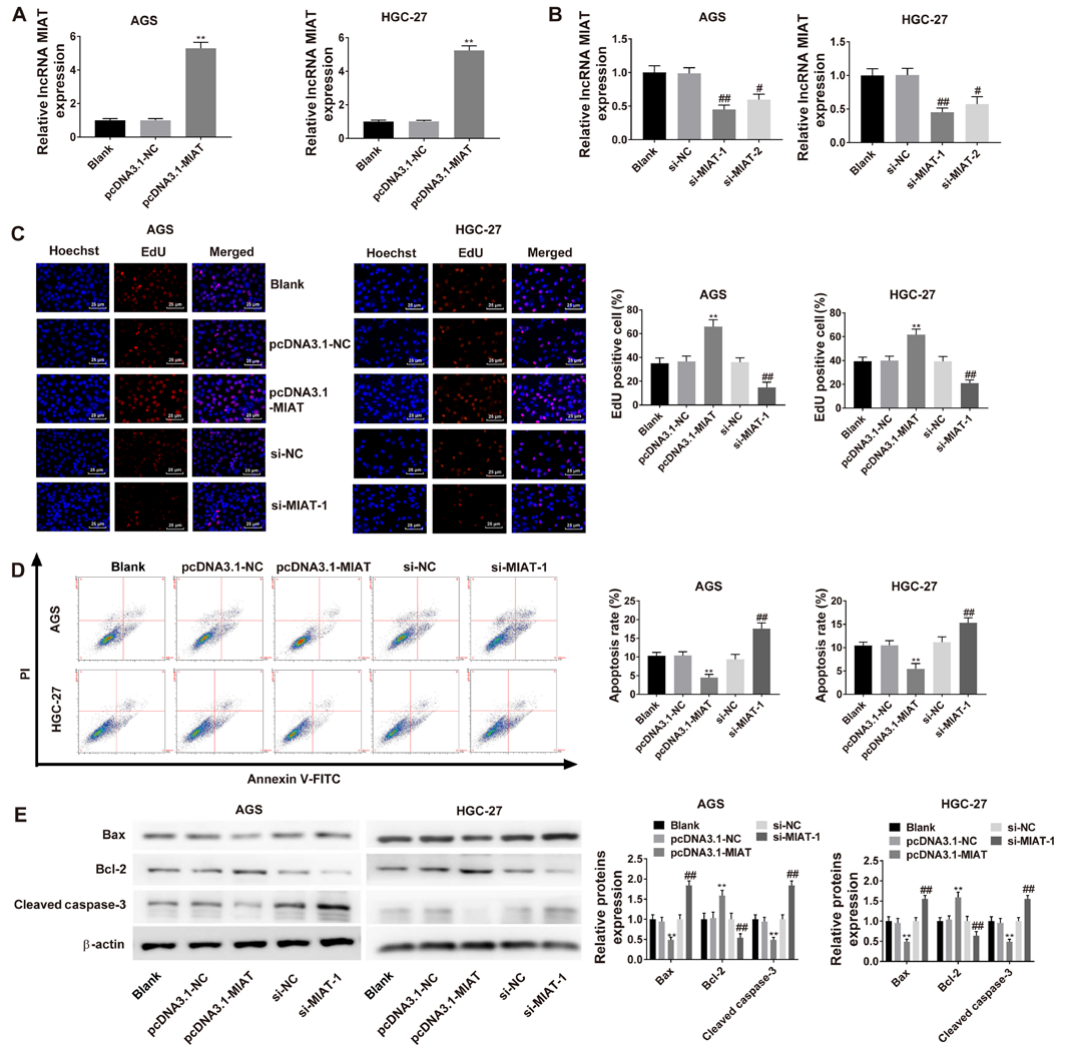
MIAT promotes GC cell proliferation by inhibiting apoptosis. (A) RT-qPCR analysis of MIAT expression in HGC-27 and AGS cells transfected with pcDNA3.1-NC or pcDNA3.1-MIAT. (B) RT-qPCR analysis of MIAT expression in HGC-27 and AGS cells transfected with si-NC, si-MIAT-1 or si-MIAT-2. (C) EdU analysis of cell proliferation in HGC-27 and AGS cells transfected with pcDNA3.1-NC, pcDNA3.1-MIAT, si-NC or si-MIAT-1. (D) Flow cytometric analyses of HGC-27 and AGS cells transfected with pcDNA3.1-NC, pcDNA3.1-MIAT, si-NC or si-MIAT-1. (E) The protein levels of Bax, Bcl-2 and cleaved caspase-3 in HGC-27 and AGS cells were measured via western blotting. n=3. **P<0.01 vs. the blank or pcDNA3.1-NC group; ^#^P<0.05, ^##^P<0.01 vs. the blank or si-NC group. GC, gastric cancer; MIAT, myocardial infarction associated transcript; si, small interfering; NC, negative control; RT-qPCR, reverse transcription-quantitative PCR; PI, propridium iodide.

Overexpression of MIAT was induced in HGC-27 and AGS cells following transfection with pcDNA3.1-MIAT (Fig. 2A). The results revealed that both si-MIAT-1 and si-MIAT-2 downregulated MIAT expression levels (Fig. 2B). Cell proliferation was significantly increased in cells transfected with pcDNA3.1-MIAT compared with cells transfected with pcDNA3.1-NC. Knockdown of MIAT significantly suppressed cell proliferation in HGC-27 and AGS cells (Fig. 2C). The apoptotic rate of HGC-27 and AGS cells was decreased by overexpression of MIAT and increased by knockdown of MIAT (Fig. 2D). The expression of Bax and cleaved caspase-3 were significantly decreased following pcDNA3.1-MIAT transfection and increased by transfection withsi-MIAT-1. By contrast, the expression of Bcl-2 was increased by pcDNA3.1-MIAT transfection and decreased by transfection with si-MIAT-1 (Fig. 2E).

#### MIAT promotes HGC-27 and AGS cell migration and invasion

**Figure 3.**
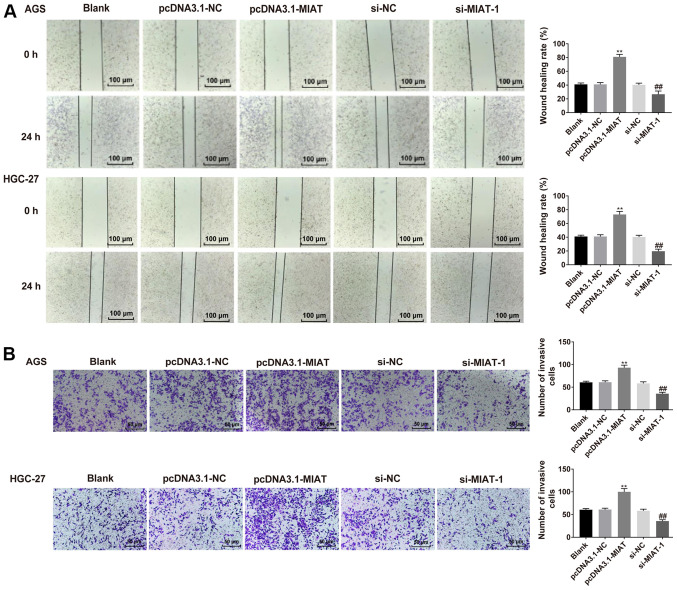
MIAT promotes GC cell migration and invasion. (A) Wound-healing analysis of cell migration in HGC-27 and AGS cells transfected with pcDNA3.1-NC, pcDNA3.1-MIAT, si-NC or si-MIAT-1. (B) Invasion assay analyses in HGC-27 and AGS cells transfected with pcDNA3.1-NC, pcDNA3.1-MIAT, si-NC or si-MIAT-1. n=3. **P<0.01 vs. the blank or pcDNA3.1-NC group; ^##^P<0.01 vs. the blank or si-NC group. GC, gastric cancer; MIAT, myocardial infarction associated transcript; si, small interfering; NC, negative control.

To determine the effect of MIAT on HGC-27 and AGS cell migration and invasion, wound healing and Transwell assays were performed. HGC-27 and AGS cell proliferation in serum-free medium was assessed via the CCK-8 assay. HGC-27 and AGS cell proliferation in serum-free medium was slightly lower compared with in medium with 10% FBS (Fig. S2). It was demonstrated that HGC-27 and AGS cells overexpressing MIAT exhibited a faster closing of the scratch wound compared with the NC group (Fig. 3A). MIAT overexpression significantly promoted cell invasion relative to the NC group. The opposite results were observed following MIAT knockdown (Fig. 3B).

#### MIAT serves as a miR-331-3p sponge in HGC-27 and AGS cells

**Figure 4.**
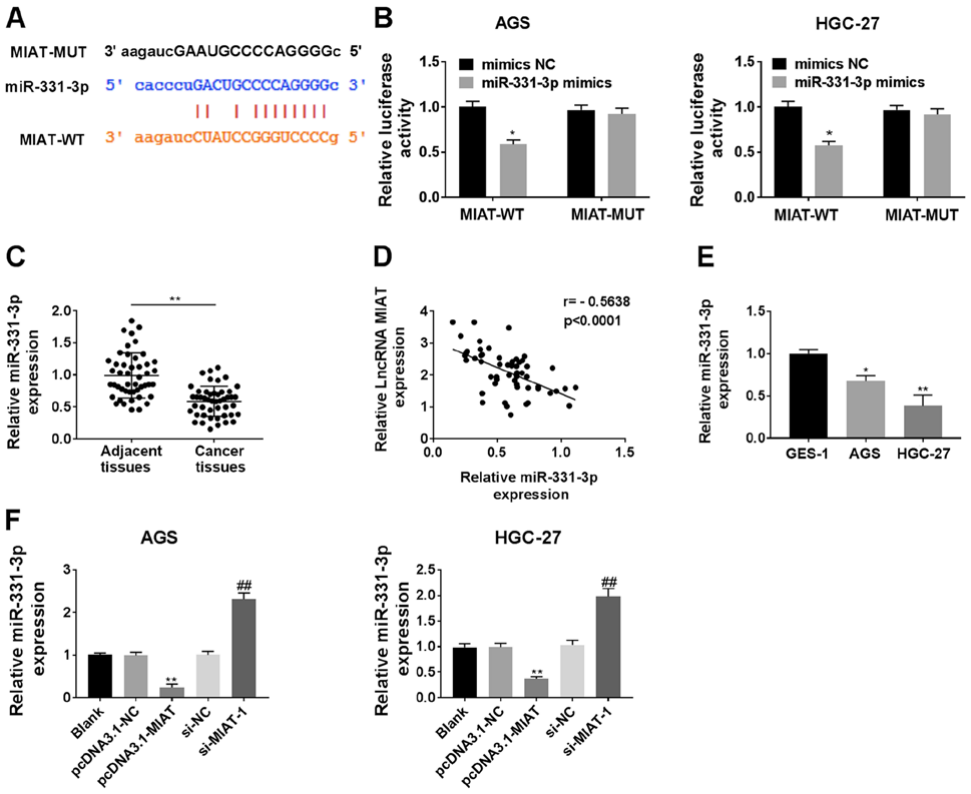
MIAT serves as a miR-331-3p sponge in GC cells. (A) The predicted binding sites between miR-331-3p and MIAT 3′UTR. (B) The luciferase activities of the MIAT-WT or MIAT-MUT reporter were detected via a dual-luciferase reporter assay. *P<0.05 vs. mimics NC/MIAT-WT. (C) miR-331-3p levels were measured cia RT-qPCR in 47 pairs of GC tissues and adjacent tissues. n=3. **P<0.01 vs. adjacent tissues group. (D) Correlation analysis between miR-331-3p and MIAT in 47 GC tissue samples. (E) miR-331-3p levels were detected via RT-qPCR in different cell lines (GES-1, HGC-27 and AGS). n=3. *P<0.05, **P<0.01 vs. the GES-1 group. (F) HGC-27 and AGS cells were transfected with pcDNA3.1-NC, pcDNA3.1-MIAT, si-NC or si-MIAT-1, followed by the measurement of miR-331-3p levels. n=3. **P<0.01 vs. the blank or pcDNA3.1-NC group; ^##^P<0 .01 vs. the blank or si-NC group. GC, gastric cancer; MIAT, myocardial infarction associated transcript; si, small interfering; NC, negative control; miR, micro RNA; RT-qPCR, reverse transcription-quantitative PCR.

Using StarBase 3.0, it was predicted that miR-331-3p had binding sites complementary to MIAT (Fig. 4A). Overexpression of miR-331-3p suppressed the luciferase activity of MIAT WT 3′-UTR in the HGC-27 and AGS cells (Fig. 4B). The expression of miR-331-3p was downregulated in GC tissues compared with adjacent tissues (Fig. 4C), and miR-331-3p expression was negatively correlated with the expression of MIAT in GC tissues (r=−0.5638; P<0.0001; Fig. 4D). miR-331-3p was also significantly decreased in HGC-27 and AGS cells compared with the GES-1 cells (Fig. 4E). Overexpression of MIAT suppressed the expression of miR-331-3p, while MIAT knockdown increased the expression of miR-331-3p in both AGS and HGC-27 cells (Fig. 4F).

#### MIAT promotes RAB5B expression via miR-331-3p

**Figure 5.**
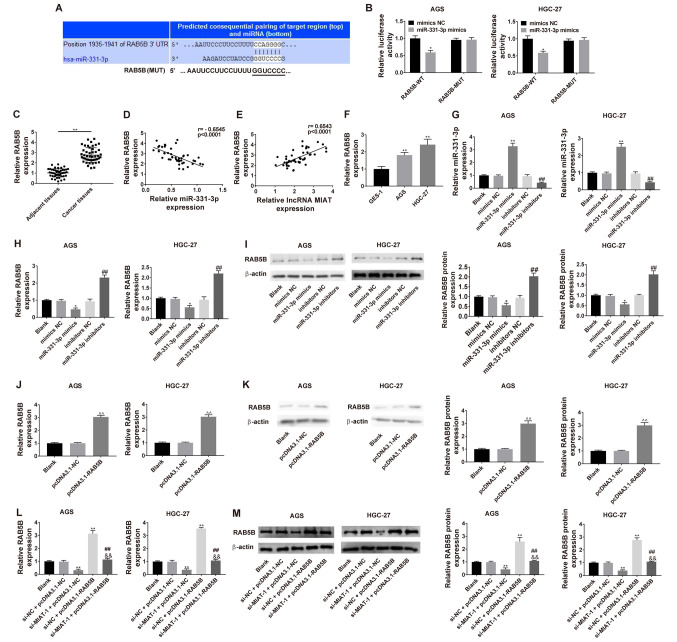
Dual-luciferase reporter system detection of the effect of miR-331-3p on RAB5B in HGC-27 and AGS cells. (A) Predicted binding between miR-331-3p and RAB5B. (B) Luciferase activities of RAB5B-WT or RAB5B-MUT reporter were detected via dual-luciferase reporter assay. *P<0.05 vs. mimics NC/RAB5B-WT. (C) RAB5B levels were measured via RT-qPCR in 47 pairs of GC tissues and adjacent tissues. (D) Correlation analysis between miR-331-3p and RAB5B in 47 GC tissue samples. n=3. **P<0.01 vs. the adjacent tissues group. (E) Correlation analysis between MIAT and RAB5B in 47 GC tissue samples. (F) RAB5B levels were detected via RT-qPCR in different cell lines (GES-1, HGC-27 and AGS). n=3. **P<0.01 vs. the GES-1 group. (G) HGC-27 and AGS cells were transfected with mimics NC, miR-331-3p mimics, inhibitors NC or miR-331-3p inhibitors, followed by the measurement of miR-331-3p level. HGC-27 and AGS cells were transfected with mimics NC, miR-331-3p mimics, inhibitors NC or miR-331-3p inhibitors, followed by the measurement of RAB5B level at the (H) mRNA and (I) protein levels. n=3. *P<0.05 vs. the blank or mimics NC group; ^##^P<0.01 vs. the blank or inhibitors NC group. (J) HGC-27 and AGS cells were transfected with pcDNA3.1-NC or pcDNA3.1-NC, followed by (K) the measurement of RAB5B level. n=3. ^^^^P<0.01, compared with the blank or pcDNA3.1-NC group. (L) Transfection efficiency in co-transfected AGS and HGC-27 cells. (M) Measurement of RAB5B level at 48 h after co-transfection. n=3. *P<0.05, **P<0.01 vs. the blank or si-NC + pcDNA3.1-NC group; ^##^P<0.01 vs. the si-MIAT-1 + pcDNA3.1-NC group; ^&&^P<0.01 vs. the si-NC + pcDNA3.1-RAB5B group. GC, gastric cancer; MIAT, myocardial infarction associated transcript; si, small interfering; NC, negative control; miR, micro RNA; WT, wild-type; MUT, mutant; RT-qPCR, reverse transcription-quantitative PCR.

Bioinformatics analysis using the TargetScan 7.2 revealed that miR-331-3p potentially binds to the 3′UTR of RAB5B mRNA, which was significantly reduced by transfection of miR-331-3p mimics (Fig. 5A and B). The expression of RAB5B was markedly higher in GC tissues and HGC-27 and AGS cell lines (Fig. 5C and F, respectively). A correlation analysis revealed that the mRNA expression of RAB5B in GC tissues was inversely correlated with miR-331-3p levels (r=−0.6545; P<0.0001) and positively correlated with MIAT levels (r=0.6543; P<0.0001) (Fig. 5D and E, respectively). The expression of miR-331-3p was upregulated in AGS and HGC-27 cells transfected with miR-331-3p mimics, whereas it was downregulated in cells transfected with miR-331-3p inhibitors (Fig. 5G). The mRNA and protein expression levels of RAB5B were downregulated in HGC-27 and AGS cells transfected with miR-331-3p mimics, whereas they were was upregulated in AGS and HGC-27 transfected with miR-331-3p inhibitors (Fig. 5H and I, respectively). The mRNA and protein expression of RAB5B was upregulated in AGS and HGC-27 transfected with pcDNA-RAB5B (Fig. 5J and K, respectively). Subsequently, the mRNA and protein expression of RAB5B were determined after transfection of si-MIAT and pcDNA-RAB5B into HGC-27 and AGS cells and it was demonstrated that the decreased expression of RAB5B in response to si-MIAT was partially reversed by RAB5B overexpression (Fig. 5L and M).

#### MIAT knockdown effects are reversed by miR-331-3p inhibitor or RAB5B overexpression in HGC-27 and AGS cells

**Figure 6.**
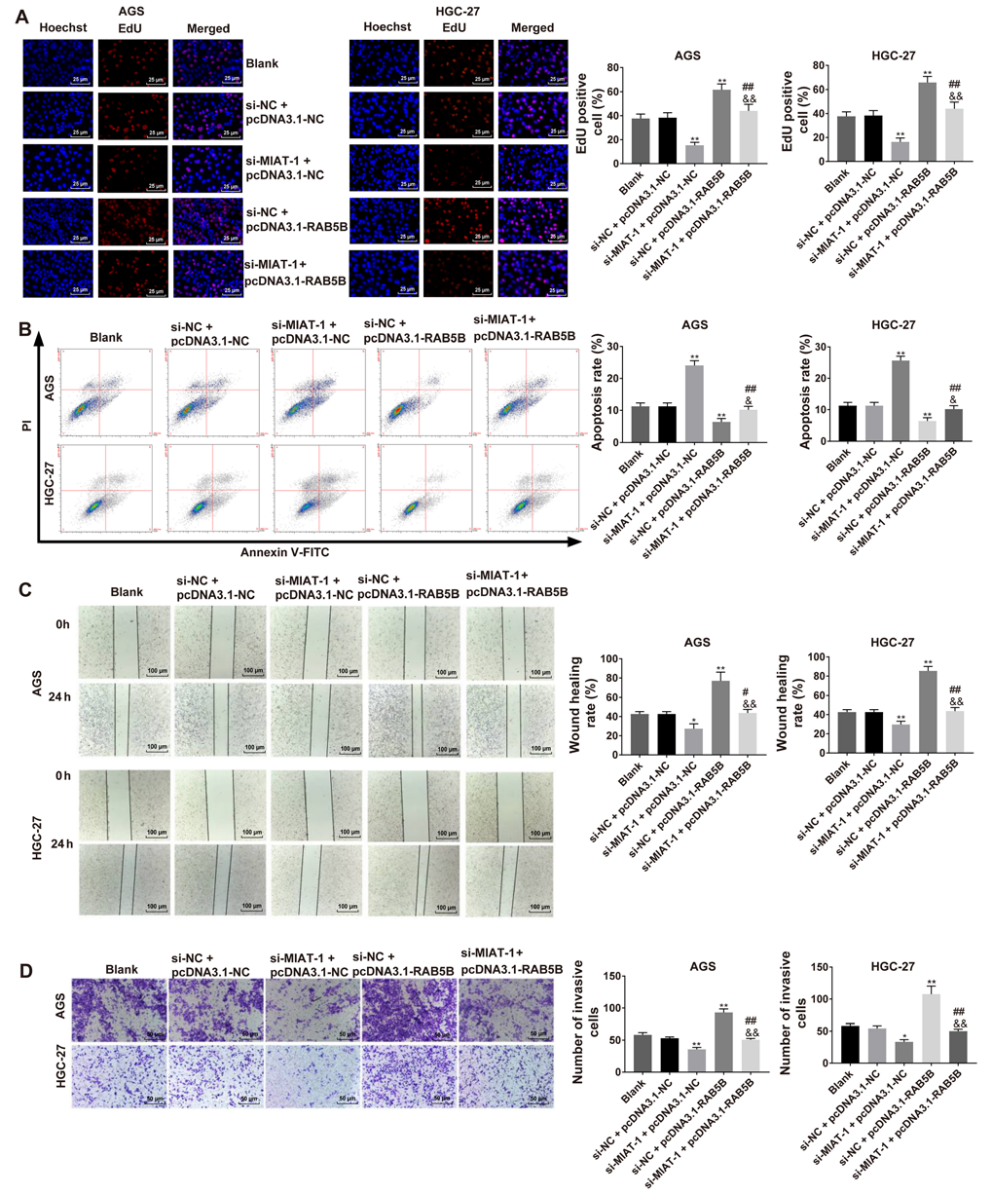
Regulatory functions of MIAT knockdown on regulating cell proliferation, apoptosis, migration and invasion were reversed by RAB5B overexpression in GC cells. (A) Cell proliferation was assessed via EdU assay. (B) Cell apoptosis was evaluated via flow cytometry. (C) Cell migration capacity was evaluated using a wound healing assay. (D) Cell invasion capacity was evaluated by the Transwell invasion assay. n=3. *P<0.05, **P<0.01 vs. the blank or si-NC + pcDNA3.1-NC group; ^#^P<0.05, ^##^P<0.01 vs. the si-MIAT-1 + pcDNA3.1-NC group; ^&^P<0.05, ^&&^P<0.01 vs. the si-NC + pcDNA3.1-RAB5B group. GC, gastric cancer; MIAT, myocardial infarction associated transcript; si, small interfering; NC, negative control; PI, propidium iodide.

The results revealed that MIAT knockdown inhibited cell proliferation (Fig. S3A) and invasion (Fig. S3B) in HGC-27 and AGS cells, while these functions were abrogated by miR-331-3p inhibitor. It was also demonstrated that MIAT knockdown inhibited cell proliferation (Fig. 6A), induced cell apoptosis (Fig. 6B), and inhibited migration (Fig. 6C) and invasion (Fig. 6D) in HGC-27 and AGS cells, while these functions were reversed by RAB5B overexpression.

#### Influence of MIAT overexpression on cell proliferation, apoptosis, migration and invasion are reversed by RAB5B knockdown in GES-1 cells

**Figure 7.**
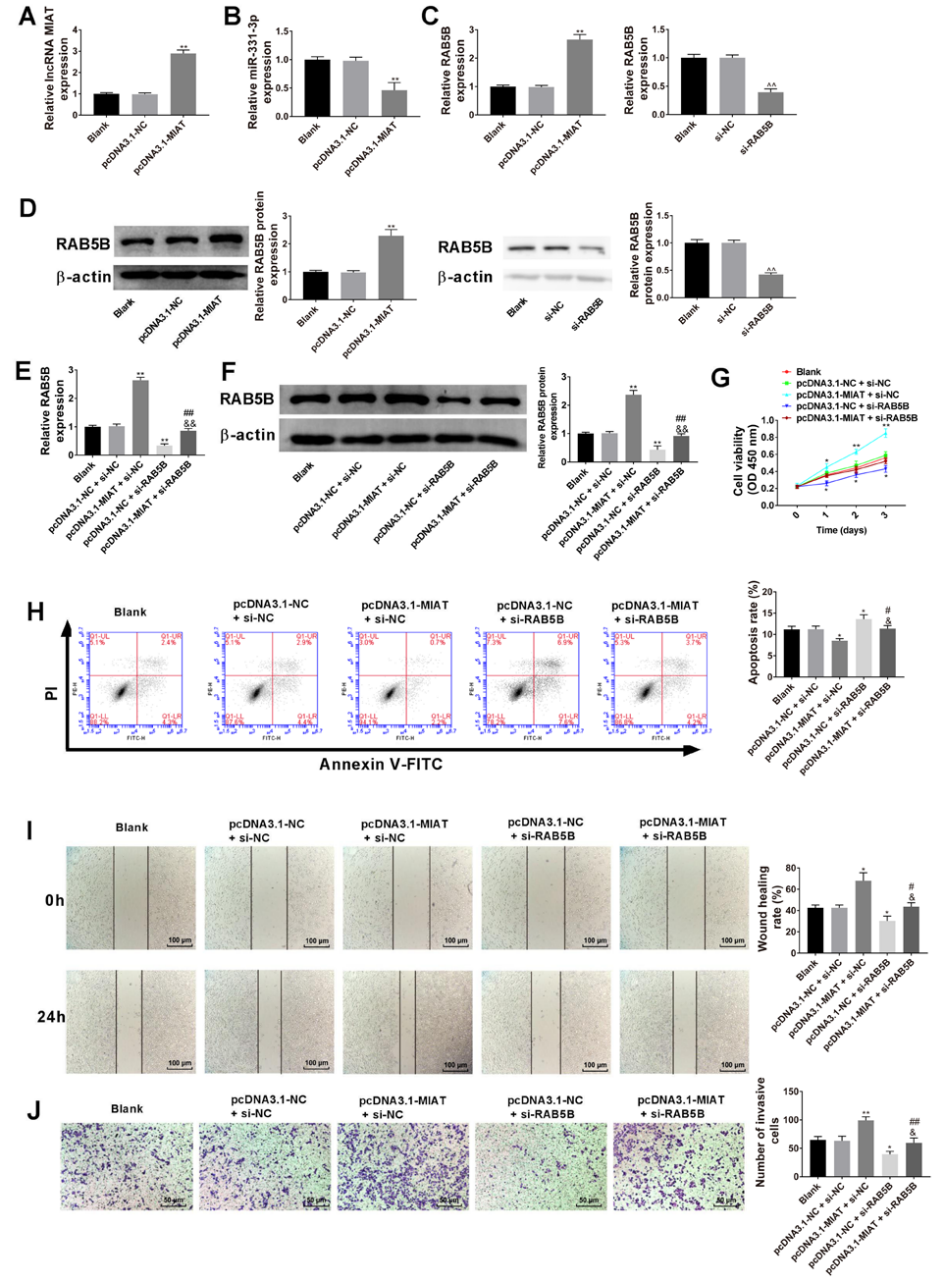
Influence of MIAT-overexpression on GES-1 cell proliferation, apoptosis, migration and invasion are reversed by RAB5B-knockdown. (A) RT-qPCR analysis of MIAT expression in the gastric epithelial mucosa cell line GES-1. (B) RT-qPCR analysis of miR-331-3p expression in GES-1 cells. (C) RT-qPCR and (D) western blot analysis of RAB5B expression in GES-1 cells, n=3. **P<0.01 vs. the blank or pcDNA3.1-NC group, ^^^^P<0.01 vs. the blank or si-NC group. (E) HGC-27 and AGS cells were transfected with pcDNA3.1-NC, pcDNA3.1-MIAT, si-NC, or si-RAB5B, followed by (F) the measurement of RAB5B level. (G) Cell proliferation ability was assessed by the Cell Counting Kit-8 assay. (H) Cell apoptosis was evaluated by flow cytometry. (I) Cell migration capacity was evaluated using a wound healing assay. (J) Cell invasion capacity was evaluated by the Transwell invasion assay. n=3. *P<0.05, **P<0.01 vs. the blank or pcDNA3.1-NC + si-NC group; ^#^P<0.05, ^##^P<0.01 vs. the pcDNA3.1-MIAT + si-NC group; ^&^P<0.05, ^&&^P<0.01 vs. the pcDNA3.1-NC + si-RAB5B group. RT-qPCR, reverse transcription-quantitative PCR; GC, gastric cancer; MIAT, myocardial infarction associated transcript; si, small interfering; NC, negative control.

Overexpression of MIAT was induced in GES-1 cells via transfection with pcDNA3.1-MIAT (Fig. 7A). The results revealed that pcDNA3.1-MIAT downregulated miR-331-3p expression (Fig. 7B). Overexpression of MIAT upregulated RAB5B mRNA and protein expression, and knockdown of RAB5B downregulated RAB5B mRNA and protein expression (Fig. 7C and D, respectively). Subsequently, RAB5B mRNA and protein expression levels were assessed after transfection of the pcDNA3.1-MIAT and si-RAB5B into GES-1 cells and it was revealed that the increased mRNA and protein expression of RAB5B in response to pcDNA3.1-MIAT could be decreased by RAB5B knockdown (Fig. 7E and F). It was also demonstrated that MIAT overexpression induced cell viability (Fig. 7G), inhibited cell apoptosis (Fig. 7H), and promoted migration (Fig. 7I) and invasion (Fig. 7J) in GES-1 cells, while these functions were significantly reversed following RAB5B knockdown.

## Discussion

lncRNAs regulate gene expression via various mechanisms, including transcriptional and post-transcriptional processing, and have extensive regulatory functions in tumour initiation and progression (23). MIAT, a recently identified oncogenic lncRNA, has been reported to be upregulated in several types of cancers, including papillary thyroid cancer (7), lung cancer (8) and acute myeloid leukemia (24). However, the detailed role and molecular mechanisms of MIAT in GC remain to be elucidated. MIAT was revealed to be upregulated in GC tissues, which is consistent with previous reports (25). In the present study, it was also demonstrated that high expression of MIAT was correlated with late TNM stage, lymphatic metastasis and a poor prognosis. Similarly, upregulation of lncRNA LINC00858 is associated with a poor prognosis in patients with GC (26). High expression of LINC00858 is positively associated with TNM stage and lymphatic metastasis (26). In the present study, functional experiments revealed that MIAT knockdown inhibited HGC-27 and AGS cell proliferation, induced GC cell apoptosis and inhibited HGC-27 and AGS cell migration and invasion. These results suggest that MIAT serves an important role in the GC tumorigenesis and metastasis.

Emerging evidence suggests that lncRNAs can serve as competing endogenous RNAs (ceRNAs) to regulate miRNAs, subsequently regulating expression of target genes (27). For instance, HLA-F-AS1 promotes colorectal cancer progression by sponging miR-330-3p to upregulate PFN1 expression (28). Exosome-transmitted lncARSR functions as a sponge of miR-34/miR-449 to induce c-MET and AXL expression and mediates sunitinib resistance in renal cell carcinoma (29). In the present study, it was revealed that MIAT shares miR-331-3p response elements with RAB5B and facilitates RAB5B expression via sponging miR-331-3p. RAB5B was experimentally validated as a genuine target of miR-331-3p. Functional inhibition of miR-331-3p effectively rescued the decreased expression of RAB5B protein that was induced by MIAT knockdown in HGC-27 and AGS cells, indicating that MIAT serves as a ceRNA. Two other targets have been reported to serve roles in gastric cancer downstream of MIAT. Knockdown of MIAT suppresses cell biological behaviours in gastric cancer via a mechanism involving the miR-29a-3p/HDAC4 axis (30). Moreover, MIAT promotes gastric cancer growth and metastasis via regulation of the miR-141/DDX5 pathway (25). The current results also extend the regulatory mechanism of MIAT function.

Recent studies have reported the involvement of miR-331-3p in cancer progression. miR-331-3p inhibits cell proliferation and induces cell apoptosis in nasopharyngeal carcinoma via targeting elF4B and blocks the PI3K-AKT signalling pathway (31). Reduced expression of miR-331-3p in ovarian cancer promotes proliferation and invasion, due to upregulation of its target RCC2 (13). Guo *et al* (15) reported that miR-331-3p suppresses GC cell growth via inhibiting E2F1. Zhao *et al* (32) revealed that miR-331-3p suppresses cell proliferation in triple-negative breast cancer cells via downregulating NRP2 (32). The current results also supported the regulatory role of miR-331-3p.

RAB5 has three isoforms (RAB5A, B and C) (33). Rab5B is a member of the Ras superfamily of small Rab GTPases (34). RAB5B is localized at the plasma membrane and early endosomes, and functions as a key regulator of vesicular trafficking during early endocytosis (35). Inhibition of RAB5/7 efficiently eliminates colorectal cancer stem cells and disrupts cancer foci (36). RAB5B expression is elevated in melanoma cells (37). RAB5B regulates cell adhesion and migration by promoting Rac1 activation and cancer cell migration (38). Kong *et al* (39) reported that RAB5B is directly downregulated by miR-130a-3p, and knockdown of RAB5B inhibits cell proliferation, migration and invasion of breast cancer cells. Wang *et al* (40) demonstrated that lncRNA-APC1 expression inhibits colorectal carcinoma cell growth, metastasis and tumour angiogenesis via suppressing exosome production through the direct binding of Rab5b mRNA. The present results further confirmed that RAB5B serves a critical role in the progression of GC.

In conclusion, the present study revealed that MIAT is upregulated in GC, which is associated with poor clinical outcomes. MIAT promotes HGC-27 and AGS cell proliferation via RAB5B. MIAT promotes RAB5B activity via sponging miR-331-3p to upregulate RAB5B expression. The present findings provide insight into the MIAT/RAB5B pathway, and indicate it as a promising potential therapeutic target in GC, suggesting important translational implications.

## Supplementary Material

Supporting Data

## Figures and Tables

**Table I.** siRNA sequences used in the present study.

Name	Sequences
si-MIAT-1	5′-GGUGUUAAGACUUGGUUUCTT-3′
si-MIAT-2	5′-ACUUCUUCGUAUGUUCGGCTT-3′
si-NC	5′-UUCUCCGAACGUGUCACGUTT-3′
miR-331-3p mimics	5′-GCCCCUGGGCCUAUCCUAGAA-3′
miR-331-3p inhibitors	5′-UUCUAGGAUAGGCCCCAGGGGC-3′

MIAT, myocardial infarction associated transcript; si, small interfering; NC, negative control.

**Table II.** Primers used for reverse transcription-quantitative PCR.

Primer names	Sequences
lncRNA-MIAT	F: 5′-GGACGTTCACAACCACACTG-3′
lncRNA-MIAT	R: 5′-TCCCACTTTGGCATTCTAGG-3′
miR-331-3p	F: 5′-GCGCCCCTGGGCCTATC-3
miR-331-3p	R: 5′-CGATGACCTATGAATTGACA-3′
RAB5B	F: 5′-TTCCTCACCCAGTCCGTTTG-3′
RAB5B	R: 5′-GCCTGTCGCTGTAGTTCCTT-3′
U6	F: 5′-CTCGCTTCGGCAGCACA-3′
U6	R: 5′-AACGCTTCACGAATTTGCGT-3′
β-actin	F: 5′-TGACGTGGACATCCGCAAAG-3′
β-actin	R: 5′-CTGGAAGGTGGACAGCGAGG-3′

lncRNA, long non-coding RNA; miR, microRNA; MIAT, myocardial infarction associated transcript; F, forward; R, reverse.

**Table III.** The association between lncRNA MIAT expression and clinicopathological variables of patients with gastric cancer.

		lncRNA MIAT expression		
			
Variables	Value (n)	High (n=23)	Low (n=24)	P-value
Sex				0.1351
Male	30	18	12	
Female	17	5	12	
Age, years				0.2124
≤60	15	5	10	
>60	32	18	14	
Tumor size, cm				0.3852
≤5	22	9	13	
>5	25	14	11	
Depth of invasion				0.1468
T1+T2	22	8	14	
T3+T4	25	15	10	
Differentiation				
Well or moderate	21	8	13	0.2443
Poor or other	26	15	11	
Lymph node involvement				0.0355^a^
Absence	18	6	12	
Presence	29	17	12	
TNM stage				0.0415^a^
I–II	26	9	17	
III–IV	21	14	7	

a P<0.05. lncRNA, long non-coding RNA; MIAT, myocardial infarction associated transcript.

## Data Availability

The datasets used and analyzed during the current study are available from the corresponding author on reasonable request.
