# Supplementary material for: Long non-coding RNA MIAT promotes gastric cancer proliferation and metastasis via modulating the miR-331-3p/RAB5B pathway
Source: Oncol Lett. 2020 Oct 13;20(6):355. doi: 10.3892/ol.2020.12219 (PMC7608069; doi:10.3892/ol.2020.12219)

Figure S1. Expression of MIAT was positively correlated with the cell proliferation and metastasis ability. (A) reverse transcription-quantitative PCR analysis of MIAT expression in AGS and HGC-27 cells. (B) AGS and HGC-27 cells proliferation ability was assessed by Cell Counting Kit-8 (CCK-8) assay. (C) AGS and HGC-27 cells metastasis ability was assessed by Transwell assay (magnification, x100). n=3. *P<0.05, **P<0.01 vs. the AGS group. GC, gastric cancer; MIAT, myocardial infarction associated transcript.

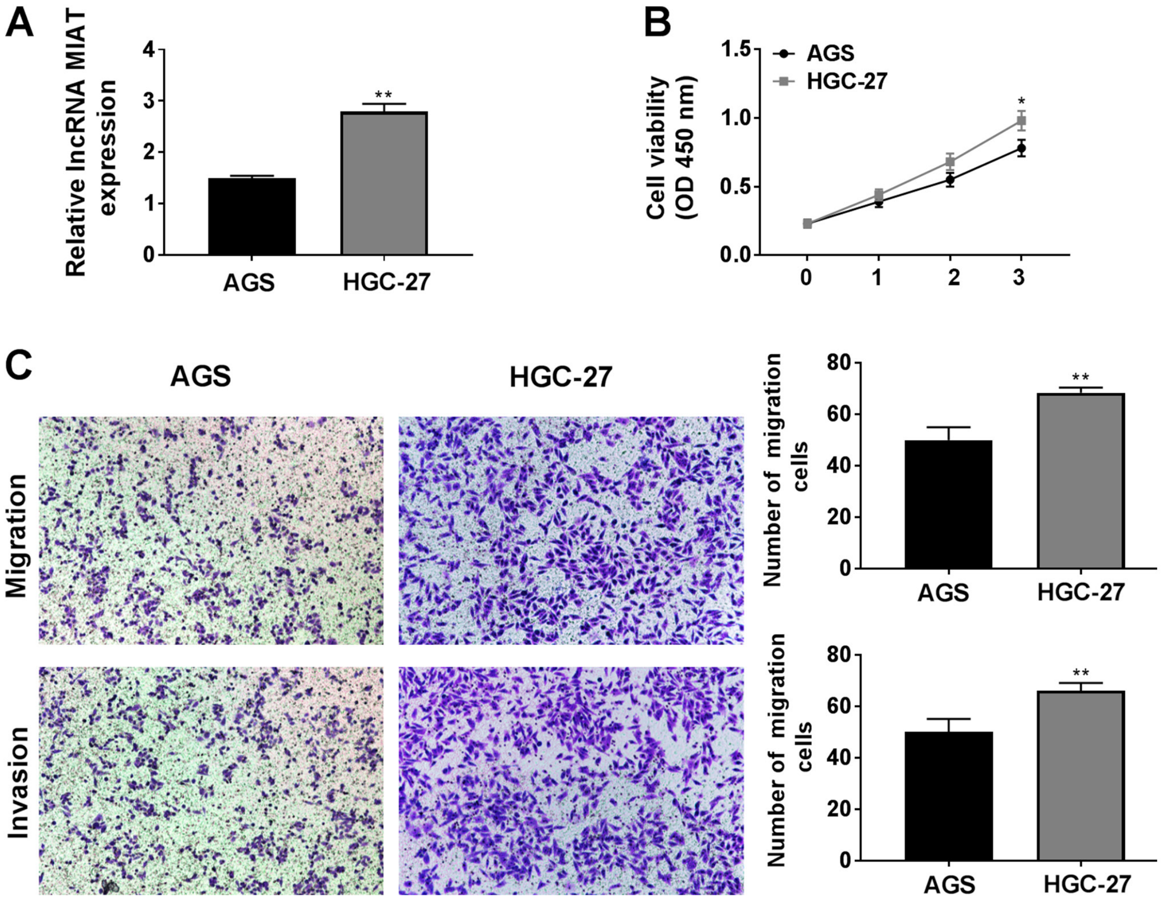

Figure S2. Cell proliferation in serum-free medium has little effect on wound healing (cell migration) and invasion assay. The AGS and HGC-27 cells without transfection (5×10^3 cells/well) were seeded into 96-well plates. The cell proliferation in serum-free medium or medium with 10% FBS was assessed by Cell Counting Kit-8 assay. n=3. **P<0.01 vs. the serum-free medium group.

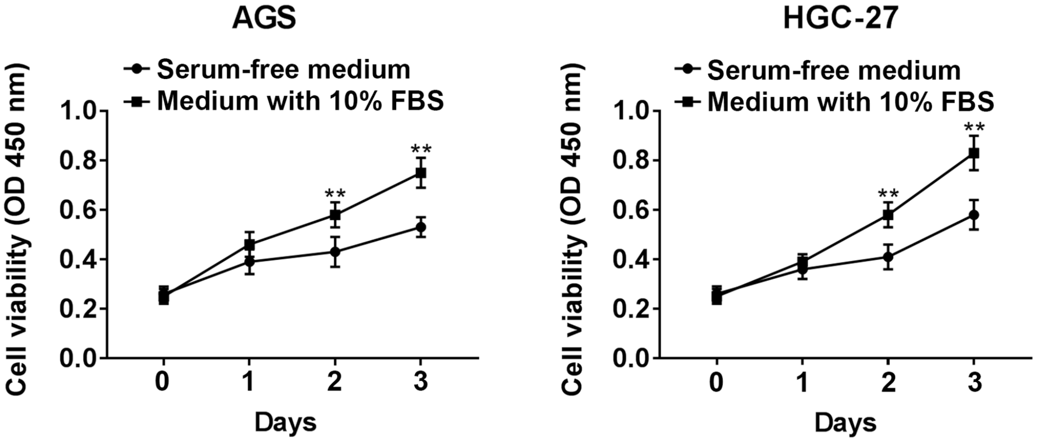

Figure S3. Functions of MIAT knockdown regulating cell proliferation and invasion were reversed by miR-331-3p inhibitor in gastric cancer cells. (A) Cell proliferation ability was assessed by Cell Counting Kit-8 assay. (B) Cell invasion capacity was evaluated by Transwell invasion assay. n=3. *P<0.05, **P<0.01 vs. the blank or si-NC + inhibitor NC group; ##P<0.01 vs. the si-MIAT-1 + inhibitor NC group; &&P<0.01 vs. the si-NC + miR-331-3p inhibitor group. MIAT, myocardial infarction associated transcript; NC, negative control; miR, microRNA; si, small interfering.

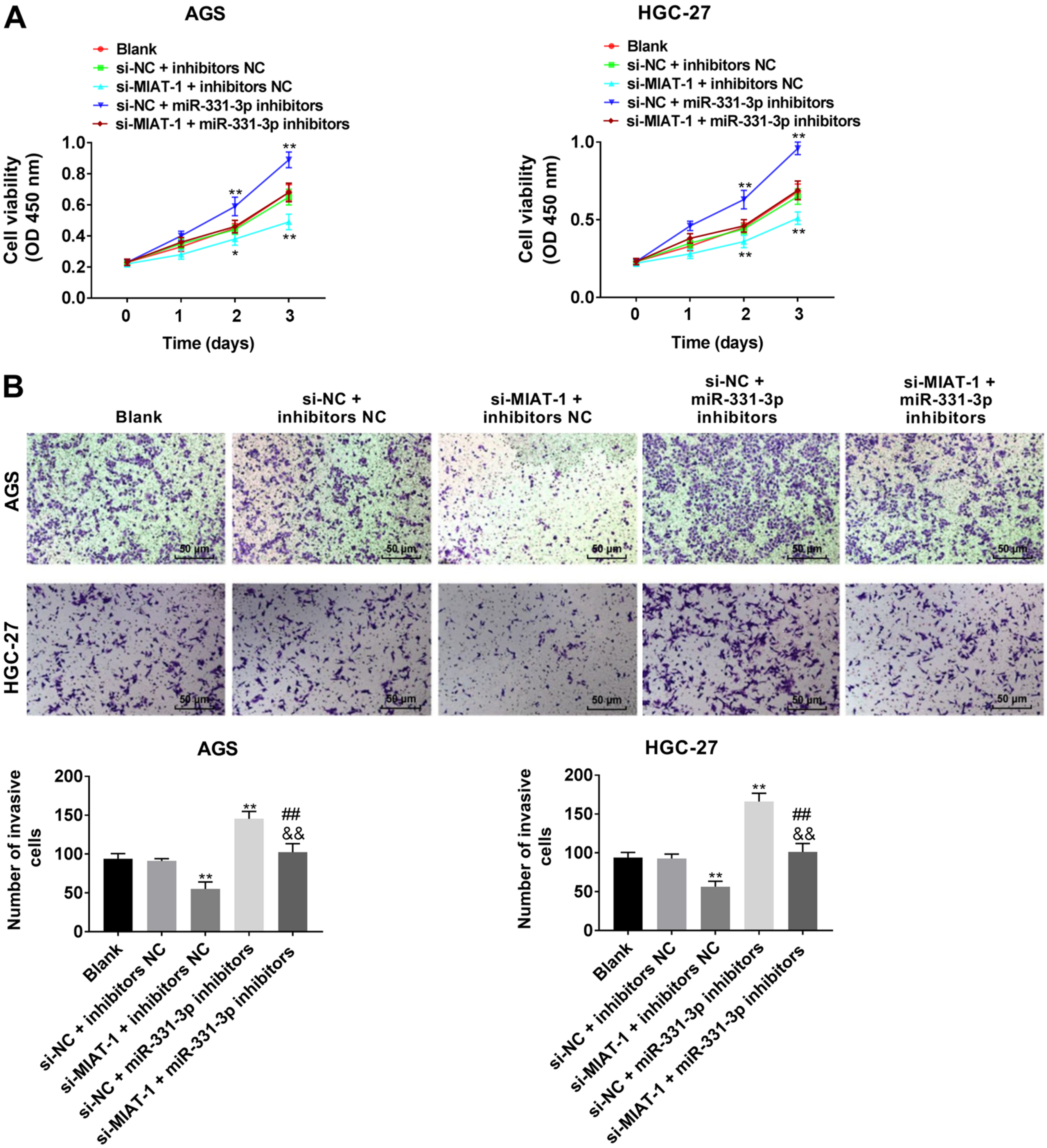

Supplement: Supporting Data [file Supplementary_Data.pdf]
